# Effect of Salicylic Acid in the Yield of Ricinine in *Ricinus communis* under Greenhouse Condition

**DOI:** 10.3390/plants10091902

**Published:** 2021-09-14

**Authors:** Carlos Eduardo Zavala-Gómez, Eloy Rodríguez-deLeón, Mamadou Moustapha Bah, Ana Angélica Feregrino-Pérez, Juan Campos-Guillén, Aldo Amaro-Reyes, José Alberto Rodríguez-Morales, Juan Fernando García-Trejo, Antonio Flores-Macias, Rodolfo Figueroa-Brito, Miguel Angel Ramos-López

**Affiliations:** 1 Biosystems Engineering Group, Division of Graduate Studies, Faculty of Engineering, Autonomous University of Queretaro, Cerro de las Campanas, S/N, Colonia Las Campanas, C.U., Santiago de Querétaro 76010, Mexico; ezavala2@gmail.com (C.E.Z.-G.); feregrino.angge@hotmail.com (A.A.F.-P.); fernando.garcia@uaq.mx (J.F.G.-T.); 2 Faculty of Chemistry, Autonomous University of Querétaro, Cerro de las Campanas, S/N, Colonia Las Campanas, Santiago de Querétaro 76010, Mexico; eloy.q22@gmail.com (E.R.-d.); moubahdia@yahoo.com.mx (M.M.B.); camposj78@hotmail.com (J.C.-G.); aldoamaro@gmail.com (A.A.-R.); 3 Nanosystems Laboratory, Division of Graduate Studies, Faculty of Engineering, Autonomous University of Querétaro, Cerro de las Campanas, S/N, Colonia Las Campanas, Santiago de Querétaro 76010, Mexico; josealberto970@hotmail.com; 4 Department of Agricultural and Animal Production, Universidad Autónoma Metropolitana-Xochimilco, Calzada del Hueso 1100, Mexico City 04960, Mexico; aflores981@gmail.com; 5 Biotic Products Development Center, National Polytechnic Institute, Calle Ceprobi No. 6, Yautepec 62731, Mexico; rfigueroa@ipn.mx

**Keywords:** *Ricinus communis*, elicitation, ricinine

## Abstract

Castor bean (*Ricinus communis*) seeds contain ricinine, an alkaloid with insecticidal and insectistatic activities. Elicitation with salicylic acid (SA) has proven to stress *R. communis* and might modify the ricinine concentration. The aim of this study was to evaluate the concentration of ricinine in the bagasse of seeds from *R. communis* elicited with exogenous SA under greenhouse conditions. Plants were grown and divided into five groups, which were sprayed with SA and drench with 50 mL 60 days after sowing with concentrations of SA (0, 100, 300, 600 and 900 µM). Clusters were mixed and separated according to the treatment, and dried. The seeds were ground, the oil was extracted by Soxhlet with hexane, and then the bagasse was extracted with methanol. Ricinine was determined by HPLC. Elicitation did not change the plant height or diameter; the control group had 9.17 µg mL^−1^ of ricinine; and the concentrations followed a hormesis curve with the peak at 300 µM of SA that had a ricinine concentration of 18.25 µg mL^−1^. Elicitation with SA might be a cost-effective technique to increase ricinine from *R. communis* bagasse.

## 1. Introduction

Castor bean plant *Ricinus communis* is a nonedible seed crop. This species is native of tropical Africa, and due to its commercial importance, is currently cultivated worldwide in countries with tropical weather such as Mexico [1]. This plant contains metabolites that include proteins, carbohydrates, minerals, albumins, ricinoleic acid, ricin, ricinine, N-demethylricinine, kaempferol-3-0-β-D-xyylopyranoside, kaempferol-3-0-β-D-glucopyranoside, quercetin-3-0-β-D-xylopyranoside, quercetin-3-0-β-D-glucopyranoside, kaempferol-3-0- β -rutinoside, and quercetin-3-0-β-rutinoside, which makes the plant functional in more than 700 industrial applications such as the production of cosmetics, paints, biofuels and pesticides [2,3,4,5].

Ricinine is one of the most important metabolites of *R. communis*: it is an alkaloid (3-Cyano-4 methoxy-N-methyl-2-pyridone), which belongs to the group of piperidine alkaloids, and depending on the concentration, it could cause emesis, nausea, and even death if ingested [6]. This alkaloid contains a cyano group in its structure, which gives its toxicity; a property that suggests its use as an insecticidal, against *Atta sexdenx rubropilosa* and against *Spodoptera frugiperda* [7,8]. It exhibits antimicrobial activity against the bacterial species *Staphylococcus aureus*, *Escherichia coli, Klebsiella pneumoniae*, *Pseudomonas aeruginosa*, and the fungal species *Candida albicans* [9]. Ricinine is present in all parts of the plant, and its production can be enhanced by nitrogen, and environmental factors such as salinity and drought [10].

An additional method to enhance the production of secondary metabolites such as alkaloids is by elicitation [11], which is the induction of the immune system of a plant by an external agent [12]. For example, Akhgari et al. [13] increased the concentration of terpenoid indole alkaloid with methyl jasmonate. Taha and Twaij [14] demonstrated an increase in phenolic compounds eliciting *R. communis* with AgNO_3_. Another common compound used in elicitation is salicylic acid (SA), which is a phenolic compound and a natural constituent of many plant species [15]; it is involved in responses to different types of stress (biotic and abiotic) and defense against pathogen attacks [16]. SA affects the oxide/reduction balance of plant cells, and induces adaptive, physiological, and morphological responses [17]. It is considered as a bioregulator of growth and has been shown to increase the production of foliar biomass in plants, roots, and fruits [18]. SA has been shown to enhance the production of alkaloids in different plant models [19].

There is no evidence of the effect of SA on the secondary metabolites of *R. communis.* However, it has been shown to increase the concentration of phenolic, flavonoid, and flavonol compounds in *Helianthus annuus* [20]; improve the yields of alkaloids and flavonoids in *Isatis tinctoria* [21]; and improve the production of benzylisoquinoline alkaloids in *Papaver armeniacum* [22].

Given the industrial importance of *R. communis* and the high availability around the world, the aim of this study was to evaluate salicylic acid in the yield of ricinine alkaloid contained in *R. communis* seeds under greenhouse conditions.

## 2. Results

### 2.1. Plant Measurement and Oil Content of R. communis

The height of the plants ranged from 51.05 cm to 58.31 cm at day 7, and 195.68 cm to 216.27 cm at day 40; the stem diameter ranged from 4.09 cm to 4.89 cm at day 7, and 8.65 cm to 9.19 cm at day 40. No statistical differences were found in the heights and diameters between the treatments (Table 1). This showed homogeneity in the growth of all plants of this study, before applying the elicitor (SA) at day 40. 

The oil content ranged from 34.89% to 50.23% between the treatments. Increments of 14% and 7.5% with respect to the control (0 µM) with the treatments of 900 µM and 600 µM were observed. The weight of 100 seeds increased 25.7% in the 900 µM treatment and 13.6% in the 600 µM treatment as compared with the control. The treatment of 100 µM decreased by 8.5%; however, this change was not statistically significant. There were no statistical differences between the treatments in the length (from 14.30 mm to 14.39 mm), width (from 9.03 mm to 9.10 mm) and thickness (from 6.60 mm to 6.66) of the seeds (Table 2).

### 2.2. Ricinine Standard Calibration Curve

**Figure 1:**
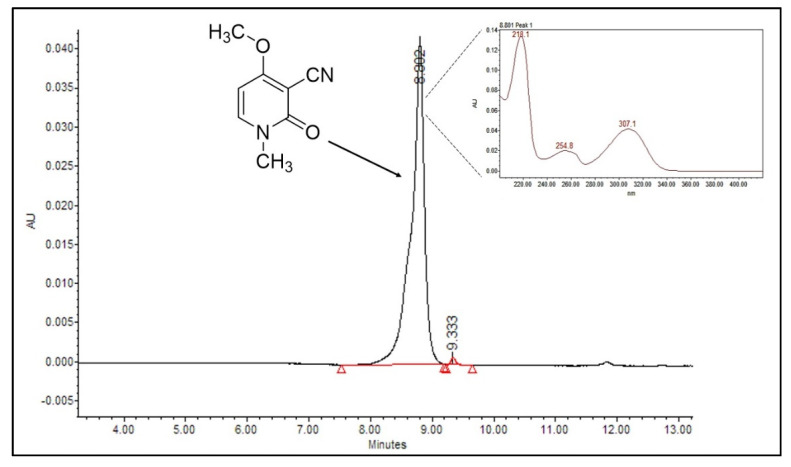
Chromatogram of ricinine standard and its absorption spectrum in UV–Vis.

**Figure 2:**
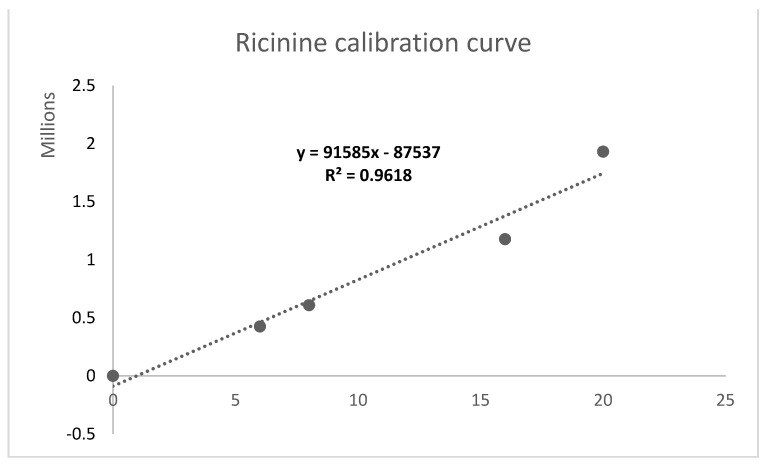
Ricinine calibration curve.

Figure 1 shows the HPLC chromatogram of the ricinine standard and its absorption spectrum in UV–Vis. The retention time of the different concentrations of ricinine standard ranged between 8.797 min and 8.814 min The areas under the curve ranged between 425,421 for the 6 µg mL^−1^ and 1,930,221 for the 20 µg mL^−1^ samples (Table 3). A high linearity of R^2^ = 0.9618 was reached, which allowed it to be used for the quantification of ricinine extracts (Figure 2). The detection and quantification limits for the calibration curve were 3.2 μg mL^−1^ and 5.3 μg mL^−1^, respectively.

### 2.3. Ricinine Quantification of Methanolic Extract

**Figure 3:**
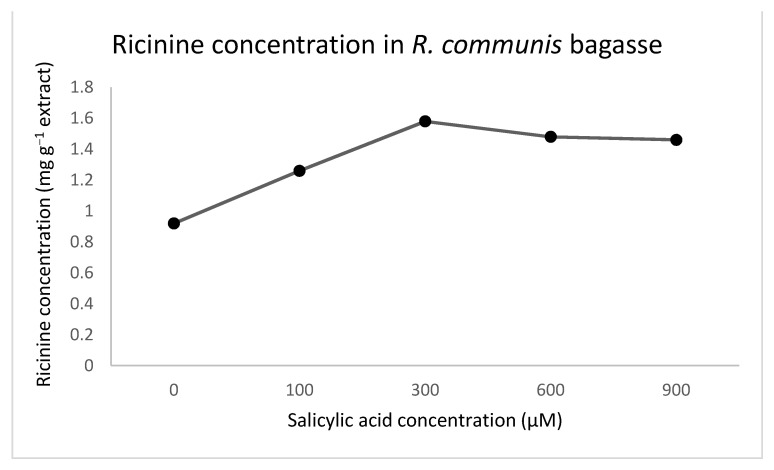
Comparison of ricinine in *R. communis* samples treated with different concentrations of SA.

**Figure 4:**
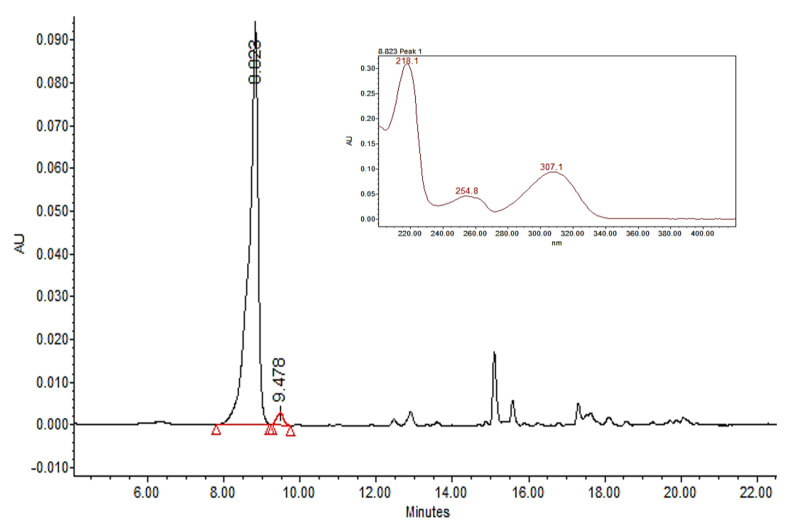
HPLC chromatogram of the sample elicited with 300 µM. salicylic acid.

All samples presented ricinine concentrations between 0.92 mg g^−1^ (0 µM) and 1.58 at mg g^−1^ (600 μM) of extract (Table 4), which is exhibited as a hormesis curve for this alkaloid using salicylic acid. The peak of this curve was at 300 µM of SA and then decreased as the concentration of SA increased, as seen in Figure 3. The chromatogram of 300 µM of SA is observed in Figure 4.

## 3. Discussion

### 3.1. Importance and Novelty of the Research

In this study, we demonstrated the effectiveness of SA as an elicitor to increase ricinine yield in *R. communis* for the first time. The highest yield was at a concentration of 300 µM of SA. 

### 3.2. Difference in Height and Diameter

In contrast to our results, other studies testing the effectiveness of SA as an elicitor found various results: Hasanuzzaman et al. [23] observed an increase of 15% over the control in the height of *Brassica campestris* plants grown in lead-amended soils (1.0 mM) and elicited with salicylic acid (0.25 mM) 30 days after sowing. However, after 45 days and at harvest, there were decreases of 35% and 26% in the heights of the plants, with respect to the control. Zamaninejad et al. [24] in *Zea mays* elicited with SA (1 mM) performed before flowering demonstrated an increase of 15% in the height and 5% stem diameter, with respect to the control. In addition, Tucuch-Haas et al. [25] in *Zea mays* treated with SA (1 µM) measured 140 days after sowing had an increase of 14% in the height and 41% of the stem diameter, with respect to the control. 

As can be seen in these studies, salicylic acid increased the height and stem diameter of the plants elicited with SA. However, in this work, no difference was found in height and stem diameter because it was not intended to increase these variables. In addition, the measurements of the plants were taken before elicitation. This study aimed to test the increase in the secondary metabolite, ricinine.

### 3.3. Effectiveness of SA as an Elicitor or Secondary Metabolites

Similar to our findings, a broad number of studies have found elicitation to be effective to increase the yield of secondary metabolites. For instance, Estaji and Niknam [26] found that *Silybum marianum* elicited with SA (1 Mm) had an increased seed oil content of 7%, as compared to the control. Safeer et al. [27] reported an increase of 14% in the seed oil content of *Helianthus annuus* elicited with SA (100 mM), with respect to the control. However, there are also studies showing conflicting evidence: Ullah and Bano [28] showed a 6% decrease in the seed oil content in *Carthamus tinctorius* elicited with SA (1 µM) with respect to the control. In this research, it was shown that the seed oil content increased by 14% with respect to the control after eliciting *R. communis* with SA (900 µM). 

For the measurements of the morphology of the seeds (length, width, and thickness) there was no significant difference between treatments. However, for the weight of 100 seeds, concentrations 600 µM and 900 µM had 25% and 14% higher weights with respect to the control, respectively; this was also reflected in the oil content.

Flores-Macías et al. [29] obtained a linearity of the calibration curve of ricinine standard of R^2^ = 0.992 by HPLC, which exhibited a high correlation with different ricinine standard concentrations (2.0, 0.2, 0.02, 0.002, and 0 mg mL^−1^). In another investigation, Wang et al. [30] reported R^2^ values of 0.9992 and 0.9988 with HPLC and LC–MS, of the standard solution of ricinine with different standard concentrations (27.7, 13.8, 6.92, 3.46, 1.73, 0.87, 0.43, 0.22, 0.11, 0.05, and 0 µg mL^−1^) and (500, 250, 125, 62.5, 31.2, 15.6, 7.8, 3.9, 1.8 and 0 ng mL^−1^), respectively. Isenberg et al. [31] determined the correlation of ricinine standard by HPLC, which showed a high linearity of R^2^ = 0.996 with different ricinine standard concentrations (200, 20.0, 0.800, and 0 ng mL^−1^). In this research, a linearity of R^2^ = 0.9618 was obtained with different ricinine standard concentrations (0, 6, 8, 16, 20 μg L^−1^); we proceeded to quantify the ricinine in the extracts.

The ricinine concentrations in the control group were similar to those reported by Nebo et al. [32] (1.3921 to 1.442 mg g^−1^) in leaves of the methanolic extract of *R. communis*. On the other hand, Flores-Macías et al. [29] found concentrations in the methanolic extracts of areal parts of *R. commuis* (894, 101, 103 and 104.2 μg mL^−1^). In this work, the concentrations of the methanolic extracts were similar to those reported by Nebo et al. [32], where the concentrations ranged from 0.92 to 1.58 mg g^−1^. However, the values for the concentrations in this study were lower than those reported by Flores-Macías et al. [29], which were between 9.17 and 18.25 μg mL^−1^.

### 3.4. Implications

Giving the high availability of wild *R. communis* in Mexico and other tropical countries and its insecticidal activity against species such as *S. frugiperda* and *A. aegypti*, it has been considered and used in insecticides [8,33,34].

These results might have implications in the commercial production of ricinine, and in the production of secondary metabolites from *R. communis*. [35]. Ricinine is an integrated pest management control alternative of biological origin that provides novel modes of action and reduces the risk of cross-resistance [36]. The use of botanical insecticides is an accessible and low-cost control alternative for farmers [37]. Furthermore, obtaining active extracts does not require complex methodologies [38]; for instance, the extracts prepared in this study were of the methanolic type and their preparation was carried out simply and quickly with the help of an ultrasonic bath. This technique has also been used and described in similar studies [39,40]. Another benefit of using biological insecticides such as ricinine is that it has specific targets; thus, it does not affect endemic fauna [41].

The ricinine extracts of this study were obtained from the bagasse resulting from the extraction of the oil, which otherwise would have been considered as toxic waste. In addition, hydrolysis of the bagasse has shown to decrease its toxicity, providing additional benefits [42].

## 4. Materials and Methods

### 4.1. Field Conditions

The study was carried out in a 432 m^2^ greenhouse at the Engineering Faculty of the Autonomous University of Querétaro Amazcala Campus in El Marques, Querétaro, Mexico (DMS latitude: 20°42‘18.576’‘ N, longitude: 100°15’57.24‘‘ W), during the seasons of autumn and winter, 2019. Weather conditions during the study were an average temperature of 27.8 °C, photoperiod of 14 h light/10 h dark, and relative humidity of 43%.

The greenhouse was divided randomly into 4 blocks, with 3 repetitions, 5 plants per experimental unit and 4 treatments of SA as an elicitor and a control distilled water.

### 4.2. Plant Materials

The seeds of the Guanajuatoil variety of *R. communis* were provided by Dr. Miguel Hernández Martínez from the National Institute of Forestry Agricultural and Livestock Research Center (INIFAP) Experimental field Celaya. 

The greenhouse was cleaned and all weeds were removed. Sowing and fertilization were carried out according to the methodology proposed by Hernández Martínez and Montes Hernández [43]: 600 seeds (2 per hole) were sown, at a depth of 30 cm, with a distance of 0.7 m between each other, across 10 cultivation lines with a separation of 1 m between lines. Sowing was carried out on 24 July 2019. Fertilization was performed by fertigation with doses of 60-40-00 NPK, with applications of 30-40-00 on the sowing day and a second application of 30-00-00 35 days after germination. Irrigation was carried out by means of a half drip every third day for a period of 30 min. Each group of plants was elicited with different concentrations of salicylic acid (100, 300, 600 and 900 µM) 60 days after sowing (21 September 2019) by foliar application, and by drench using a fumigation tank. The control plants received the same treatment with distilled water.

The clusters were harvested and sun-dried for 2 weeks, and then placed in a solar dryer for one more week. Finally, the seeds were extracted from the clusters.

### 4.3. Plant and Seed Measurements

The stem diameters and heights of the plants were measured at 7 and 40 days after sowing with a measuring tape (Truper H-1766, Jilotepec, State of México, Mexico). The stem diameter was measured at ground level and the height was measured from ground level to the highest point of the plant. After harvesting with a vernier (Steren, Azcapotzalco, Mexico City, Mexico), a random sample of 100 seeds was collected [44], and the weight, seed length, seed width and seed thickness were measured. 

### 4.4. Oil Extraction

The oil was extracted by taking a composite sample of 30 g seeds per treatment and block, from which they were taken and weighed on an analytical balance (Ohaus PA323C, Melrose, MA, USA) Afterwards, they were ground and inserted into a Soxhlet extractor connected to a 500 mL flask containing 250 mL of n-hexane. The extraction was conducted at 55 °C, the boiling temperature of technical-grade n-hexane (Sigma-Aldrich, Toluca, State of México, México). During the extraction, an ice bath was used to control the temperature. After the extraction, the samples were centrifuged and separated into layers to remove the solvent with a rotary evaporator: IKA RV 10 basic (Staufen im Breisgau, BW, Germany).

### 4.5. Sample Preparation

The methanolic extract of *R. communis* was obtained from the bagasse of the seeds after the oil extraction: 10 g of bagasse was placed into a 250 mL Erlenmeyer flask with 100 mL of reagent-grade methanol (Sigma-Aldrich, Toluca, State of Mexico, Mexico). The mixture was placed into ultrasonic bath [Elmasonic S 40 (H) (Singen, BW, Germany)] for one hour at 50 °C. Then, the extract was filtered by gravity into a funnel with a Whatman No 3 filter (Schenectady, NY, USA). Finally, the solvent was eliminated by using a IKA RV 10 basic (Staufen im Breisgau, BW, Germany) rotary evaporator.

### 4.6. Ricinine Quantification in R. communis Bagasse

HPLC analyses were performed using Waters equipment (Milford, MA, USA), Alliance model, composed of a quaternary pump model e2695 multi-solvent delivery system and a 2998 Diode Array Detector (DAD), using a C_18_ column Agilent Zorbax (5 μm, 150 × 4.5 mm) (Santa Clara, CA, USA). The mobile phase was composed of aqueous acetic acid 0.0125N and acetonitrile (CH_3_CN) in a linear gradient (as shown in Table 5) at a flow rate of 1.0 mL min^−1^. The run time was of 25 min, the volume of the injection was 20 μL and the ricinine was detected at 310 nm. Data acquisition and processing of chromatographic information were performed by Empower 3 Software (Milford, MA, USA).

For the quantification of ricinine, the calibration curve was obtained using the following concentrations (µg mL^−1^): 0, 6, 8, 16, and 20. The areas under the curves (y) obtained for each standard were plotted versus the concentrations used (x), and a linear correlation was established as y = mx + b. The amounts of ricinine were calculated by interpolation in the calibration curve. The detection limit and quantification limit were determined by the least-squares method.

### 4.7. Statistical Analysis

Descriptive analyses of the main characteristics of the plants treated with different concentrations of SA were performed. Analysis of covariance (ANCOVA) with a post hoc Tukey test were performed to assess differences between the treatments. Statistical significance was set at (*p* < 0.05).

## 5. Conclusions

In this research, it was found that the combination of the elicitation techniques of foliar application and drench with SA was found to improve effectively ricinine concentrations in *R. communis* plant seeds. Additionally, the hormesis point of salicylic acid was found at 300 µM to increase the concentration of this alkaloid, and start decreasing at 600 µM and having the point of least concentration at 900 µM. Given the industrial importance of ricinine, SA might be a cost-effective solution to increase its yield in *R. communis* seeds.

## Figures and Tables

**Table 1:** Height of stem diameters of *R. communis* plants before treatment with salicylic acid at days 7 and 40 after sowing.

Treatment	Height at 7 Days (cm)	Height at 40 Days (cm)	Stem Diameter at 7 Days (cm)	Stem Diameter at 40 Days (cm)
900 µM	58.21 ± 16.06 a	195.68 ± 26.92 a	4.36 ± 0.81 a	8.84 ± 0.76 a
600 µM	51.05 ± 11.15 a	216.27 ± 16.77 a	4.09 ± 0.65 a	8.83 ± 0.33 a
300 µM	57 00± 9.44 a	210.64 ± 27.09 a	4.42 ± 0.95 a	9.11 ± 0.82 a
100 µM	54.70 ± 8.38 a	209.39 ± 23.22 a	4.63 ± 0.71 a	9.19 ± 0.88 a
0 µM	58.31 ± 13.80 a	207.38 ± 26.58 a	4.89 ± 0.51 a	8.65 ± 0.71 a

The results are the average of 60 plants ± standard deviation. Different letters indicate the statistical difference Tukey test, *p* = 0.05.

**Table 2:** Oil content, weight of 100 seeds and morphological characteristics of castor seeds of plants treated with salicylic acid.

Trat	Oil Content (%)	Weight of 100 Seeds (g)	Seed Length (mm)	Seed Width (mm)	Seed Thickness (mm)
900 µM	50.23 ± 1.55 a	24.70 ± 2.89 a	14.37 ± 0.13 a	9.10 ± 0.09 a	6.66 ± 0.06 a
600 µM	43.75 ± 2.36 b	22.33 ± 3.12 ab	14.23 ± 0.12 a	9.03 ± 0.14 a	6.64 ± 0.07 a
300 µM	38.11 ± 0.78 c	21.50 ± 1.15 bc	14.37 ± 0.12 a	9.08 ± 0.10 a	6.64 ± 0.06 a
100 µM	34.89 ± 5.98 c	17.97 ± 1.58 d	14.30 ± 0.10 a	9.10 ± 0.06 a	6.60 ± 0.06 a
0 µM	36.23 ± 4.17 c	19.65 ± 2.10 cd	14.39 ± 0.30 a	9.07 ± 0.08 a	6.65 ± 0.11 a

Trat = Treatment. The data of oil content are the average of 3 measurements. Seed length, width, and thickness are the average of the measure of 100 seeds. Different letters indicate the statistical difference Tukey test, *p* = 0.05.

**Table 3:** Ricinine concentration and area under the curve of ricinine standard.

[µg mL^−1^]	Retention Time (minutes)	Area under the Curve
20	8.814	1,930,221
16	8.804	1,177,116
8	8.797	608,784
6	8.802	425,421
0	0	0

**Table 4:** Ricinine concentration and area under the curve of the methanolic extracts of *R. communis* plants treated with salicylic acid.

Trat	Weight Sample (mg)	Final Volume (mL)	Area under the Curve	Retention Time (minutes)	Concentration (µg mL^−1^)	Concentration (mg g^−1^ Extract)
900 µM	11.5	1	1,449,959	8.790	16.78	1.46 ± 0.08
600 µM	10.2	1	1,301,895	8.739	15.17	1.48 ± 0.05
300 µM	11.5	1	1,584,532	8.823	18.25	1.58 ± 0.07
100 µM	10.2	1	1,091,388	8.868	12.87	1.26 ± 0.06
0 µM	10.0	1	752,871	8.926	9.17	0.92 ± 0.05

The data of concentration is the average of 3 measures *±* standard deviation.

**Table 5:** Elution gradient for ricinine analysis.

Time (minutes)	Acetic Acid Solution 0.0125N	Acetonitrile
0	95%	5%
2	95%	5%
5	85%	15%
20	50%	50%
25	95%	5%

## Data Availability

Not applicable.
